# Local NGF and GDNF levels modulate morphology and function of porcine DRG neurites, *In Vitro*

**DOI:** 10.1371/journal.pone.0203215

**Published:** 2018-09-27

**Authors:** Andreas Klusch, Christian Gorzelanny, Peter W. Reeh, Martin Schmelz, Marlen Petersen, Susanne K. Sauer

**Affiliations:** 1 Department of Experimental Pain Research, Medical Faculty Mannheim, University of Heidelberg, Mannheim, Germany; 2 Department of Dermatology and Venereology, University Hospital Hamburg-Eppendorf, Hamburg, Germany; 3 Institute of Physiology and Pathophysiology, Friedrich-Alexander University Erlangen-Nürnberg, Erlangen, Germany; University Hospital Wurzburg, GERMANY

## Abstract

Nerve terminals of primary sensory neurons are influenced by their environment through target derived trophic factors, like nerve growth factor (NGF) or glial cell line-derived neurotrophic factor (GDNF). In mice, subpopulations of DRG neurons express receptors either for NGF or GDNF and therefore differentially respond to these neurotrophic factors. We probed neurite endings from porcine DRG neurons cultured in either NGF or GDNF and examined their shape, elongation and stimulus-evoked CGRP release. A compartmentalized culture system was employed allowing spatial separation of outgrown neurites from their somata and use of different growth factors in the compartments. We show that neurites of GDNF cultured somata extend into lateral compartments without added growth factor, unlike neurites of NGF cultured ones. Neurites of NGF cultured somata extend not only into NGF- but also into GDNF-containing compartments. GDNF at the site of terminals of NGF responsive somata led to a strong neurite arborization and formation of large growth cones, compared to neurites in medium with NGF. Functionally, we could detect evoked CGRP release from as few as 7 outgrown neurites per compartment and calculated release per mm neurite length. CGRP release was detected both in neurites from NGF and GDNF cultured somata, suggesting that also the latter ones are peptidergic in pig. When neurites of NGF cultured somata were grown in GDNF, capsaicin evoked a lower CGRP release than high potassium, compared to those grown in NGF. Our experiments demonstrate that the compartmented culture chamber can be a suitable model to assess neurite properties from trophic factor specific primary sensory neurons. With this model, insights into mechanisms of gain or loss of function of specific nociceptive neurites may be achieved.

## Introduction

Peripheral endings of dorsal root ganglion (DRG) neurons not only respond to appropriate stimuli, but are also influenced by target derived growth factors. Most of the DRG neurons project into the skin, where their target tissue comprises non-neuronal cells such as keratinocytes, fibroblasts and vascular smooth muscle cells. Moreover, the nerve endings are accompanied by Schwann cells, the glia cells of the peripheral nervous system. All these cells can secrete a variety of growth factors, like nerve growth factor (NGF), a member of the neurotrophic factor family, or glial cell-line derived factor (GDNF), a member of the transforming growth factor ß superfamily [1,2]. Multiple functions of these growth factors during neuronal development are well established, like survival, neurite outgrowth, and pathfinding [3]. Postnatally, DRG neurons no longer require the growth factors NGF and GDNF for their survival, but *in vivo* experiments have shown a significant role of both factors in the differentiation of nociceptive functions [4–6]. After nerve injury and under inflammatory conditions, NGF and GDNF are involved in neurite sprouting and branching. Furthermore, they are potent regulators of nociceptive transcriptional functions of DRG neurons such as expression of various pain-related molecules like calcitonin gene-related peptide (CGRP) and the transient receptor potential vanilloid 1 (TRPV1) [7,8].

In DRG neurons, many of the biological responses to NGF and GDNF are mediated by their binding to specific membrane receptors. NGF binds to the receptor tyrosin kinase TrkA [9]; GDNF binds to specific members of the GDNF family receptor alpha (GFRα) with subsequent signaling through activation of the tyrosin kinase c-Ret [10,11]. These signals are retrogradely communicated to the neuronal nuclei, where they control the biological responses through their influence on gene expression [10].

In mice, there are at least two groups of DRG neurons with unmyelinated axons which can be differentiated, based on distinct growth factor responsiveness and their anatomical projection areas [12,13]. One group signals through NGF/trkA and is mainly nociceptive-peptidergic, the other one signals through GDNF/c-RET and is nociceptive-non (or less)-peptidergic. In the skin, both their fibers branch perpendicularly towards the epidermis and ramify within different layers [14,15]. Centrally, they each terminate in distinct but overlapping regions of the superficial dorsal horn of the spinal cord [16,17]. The difference in projection areas between trkA- and c-Ret-expressing nociceptor populations suggests distinct roles in nociceptive transmission [12,18].

Under inflammatory conditions, growth factor composition of the environment of DRG neuron terminals can alter [19]. The defined morphological and functional consequences are not well understood. In *in vivo* experiments, a local control of the growth factor condition is challenging and a subgroup-specific investigation of their terminals is hampered by their small diameter and lack of experimental accessibility. Thus, isolated somata of DRG neurons are often used as model for their terminals [20]. However, there are differences between sensory endings and somata, such as through local protein biosynthesis, voltage-gated ion channel distribution, or TrkA signaling pathway [21].

*In vitro*, the spatial separation between DRG somata and their terminals can be modelled by a compartmentalized culture chamber [22,23]. It provides the possibility to define variables, like growth factors in the medium, separately for somata and their terminals. Techniques to investigate sensory properties of nociceptive terminals, e.g. the skin-nerve preparation, offer the possibility to record from functionally characterized cutaneous afferents [24,25]; however, an attribution of the sensory properties to different nociceptor populations with respect to the expression of NGF and GDNF receptors is difficult to achieve. In the present study, we used the Campenot chamber to culture porcine DRG somata either in NGF or GDNF. Their outgrown neurites in the adjacent compartments were subjected to these growth factors in different combinations and were investigated under morphological and functional aspects. We investigated neurite outgrowth, ending shape and chemically evoked CGRP release. The reasons to choose the pig as animal were twofold: (i) In the study of peripheral nociceptors, porcine skin has been used as a model because of its similarity to human skin. For example, the axon-reflex induced vasodilatation in skin is mediated by silent nociceptors like in man, unlike in rodents [26–28]. Moreover, single-fibre recording results from nociceptive sensory neurons in pig resemble more closely those in man than in rodents [29]. Recently, pig skin was utilized for evaluating topical analgesics for its similarity to human skin [30]. (ii) Technically, the compartmented Campenot chamber proved itself to be an ideal model for investigation of porcine DRG neurons with respect to outgrowth properties of neurites and high yield of culture chambers [31, 32].

## Experimental procedures

### Animal preparation

Dorsal root ganglia (DRG) from all levels of the spinal cord were removed post-mortem from 22 male piglets (*Sus scrofa domesticus*, supplied by a University of Heidelberg approved breeder) ranging in age from P9 to P14. Animals were sacrificed on the day of their delivery. Procedures were performed as described previously [33]. In brief, piglets were initially sedated with intramuscular azaperone (Janssen-Cilag GmbH Neuss, Germany; 28 mg/kg) and ketamine (Essex Pharma GmbH, Munich, Germany; 70 mg / kg) and subsequently killed with a lethal dose of intracardial pentobarbital (20 mg / kg). The spine was removed, cleaned and stored in cold PBS (Sigma-Aldrich, Seelze, Germany). Ethical approval for experimental procedures was issued by the Ethics committee of the regional government (Karlsruhe, Baden-Wuerttemberg, Germany).

### Isolation and culture of DRG neurons

Somata from DRG neurons were isolated similar as described previously [31]. Briefly, harvested ganglia were freed mechanically from connective tissue and incubated at 37°C for 110 min in DMEM containing gentamicin and collagenase (Invitrogen, Life Technologies, Schwerte, Germany). Then, ganglia were rinsed in PBS devoid of Ca^2+^ and Mg^2+^ and incubated for 8 min at 37°C in trypsin (Sigma-Aldrich, Seelze, Germany). Ganglia were placed in a mixture of DMEM containing gentamicin and supplemented Ham’s F12 (Gibco, Life Technologies, Schwerte, Germany), see below, and triturated with a fire-polished siliconized Pasteur pipette. The suspension was subsequently transferred to 10% Percoll solution and centrifuged (740 RZB for 5 min). The supernatant, containing connective tissue was removed and the pellet containing somata was resuspended in DMEM and centrifuged (170 RZB for 5 min). Culture medium was Ham’s F12 supplemented with 10% heat-inactivated horse serum (Gibco, Life Technologies, Schwerte, Germany), 2 mM L-glutamine, 100 U/ml penicillin and 10 μg/ml streptomycin.

### Culture chamber and media

**Fig 1:**
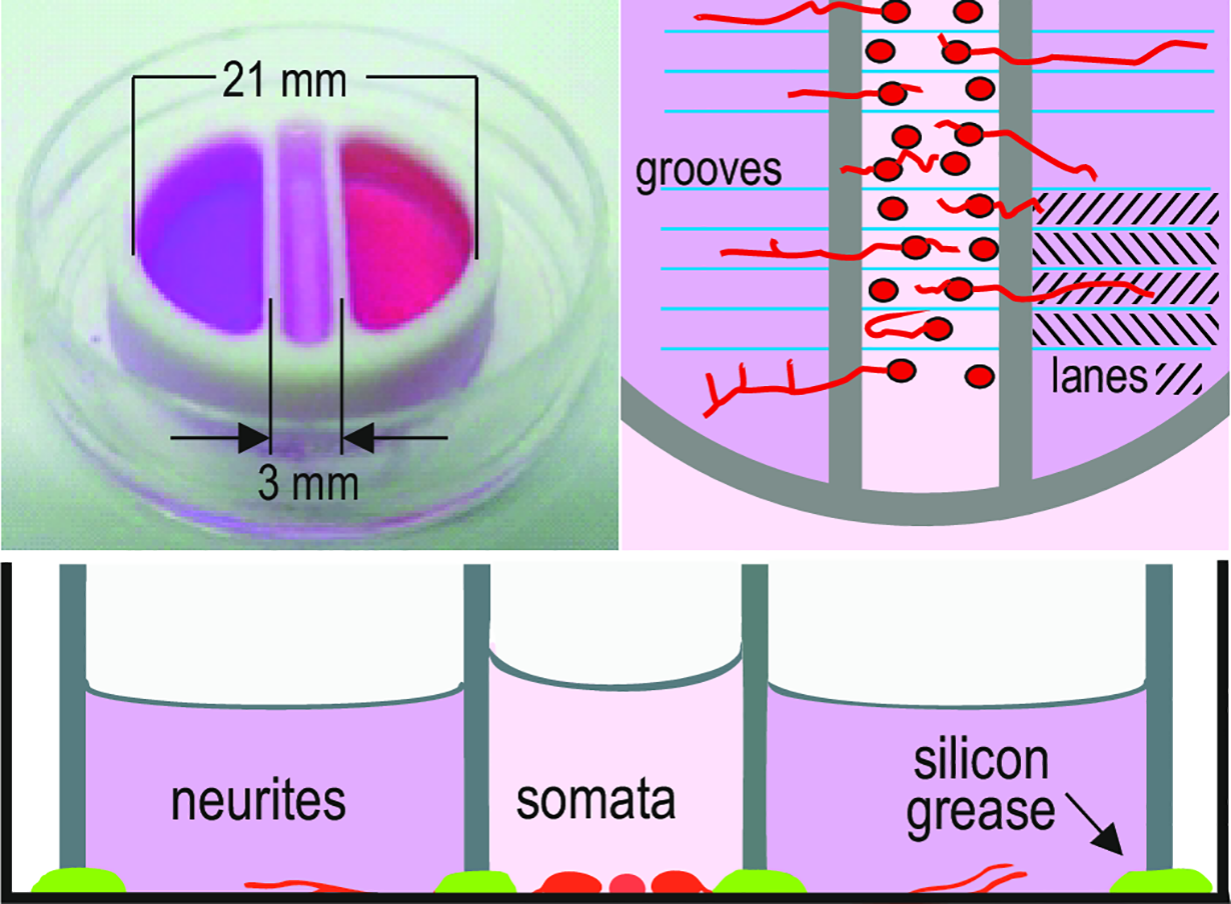
Schematic layout of culture chamber as top and side view and photograph.

A three-compartment Campenot chamber [22] with an inner diameter of 21 mm was used. Dividing walls between compartments were 1 mm thick. To accomplish outgrowth of neurites from the somata compartment into the lateral compartments, 45 parallel grooves 300 μm apart were scratched into the plastic substrate of the petri dishes to which the chambers were attached with high-viscosity silicone grease (Fig 1). Two small stripes of methyl cellulose solution (2%) were applied to the dish bottom at the locus of the two central dividing walls before attaching the chamber. The jelly provided by the methyl cellulose underneath the dividing wall and the silicone grease offers a micro-environment for the communication between the compartments. In the lateral compartments, outgrown neurites grow between two grooves and do not cross them. In the following, we denote the interspace between two grooves as a”lane“.

Dissociated cells were seeded with a density of approx. 50/mm^2^ into the central lumen of the chamber. From one piglet, up to 45 chambers could be loaded. Culture medium was supplemented Ham`s F12 with either rhß-NGF (Calbiochem, Schwalbach, Germany) or rh-GDNF (R&D System, Minneapolis, USA). Culture medium for the neurites in the lateral compartment was supplemented Ham`s F12 with either rhß-NGF or rh-GDNF with anti-NGF (Sigma-Aldrich, Seelze, Germany) or without added growth factors. Growth factor combinations listed in the following Table 1 were used as indicated.

**Table 1:** Growth factor combinations and concentrations.

*Culture condition(abbreviation)*	*Central compartment (concentration)*	*Lateral compartments* *(concentration)*
Ø-N-Ø, control	NGF (50 ng/ml)	no growth factor
N-N-N	NGF (50 ng/ml)	NGF (100 ng/ml)
G-N-G	NGF (50 ng/ml)	GDNF (100 ng/ml) anti-NGF (2 μl/ml)
Ø-G-Ø	GDNF (50 ng/ml)	no growth factor
G-G-G	GDNF (50 ng/ml)	GDNF (100 ng/ml) anti-NGF (2 μl/ml)

Cells were kept in culture at 37°C in a 5% CO_2_ humidified atmosphere and half of the medium was replaced every 2–3 days.

After experiments, leak-tightness between compartments was tested. For this, trypan blue solution (0.4%, 50 μl, Sigma-Aldrich, Seelze, Germany) was applied to the central compartment. After 16–22 h, fluid samples of all compartments were taken and photometrically measured. The optical density was compared to a series of standard trypan blue dilutions with a cut-off of 1/3000 dilution. Only chambers meeting this standard were included in the data set.

### Morphology of endings and length of neurites

**Fig 2:**
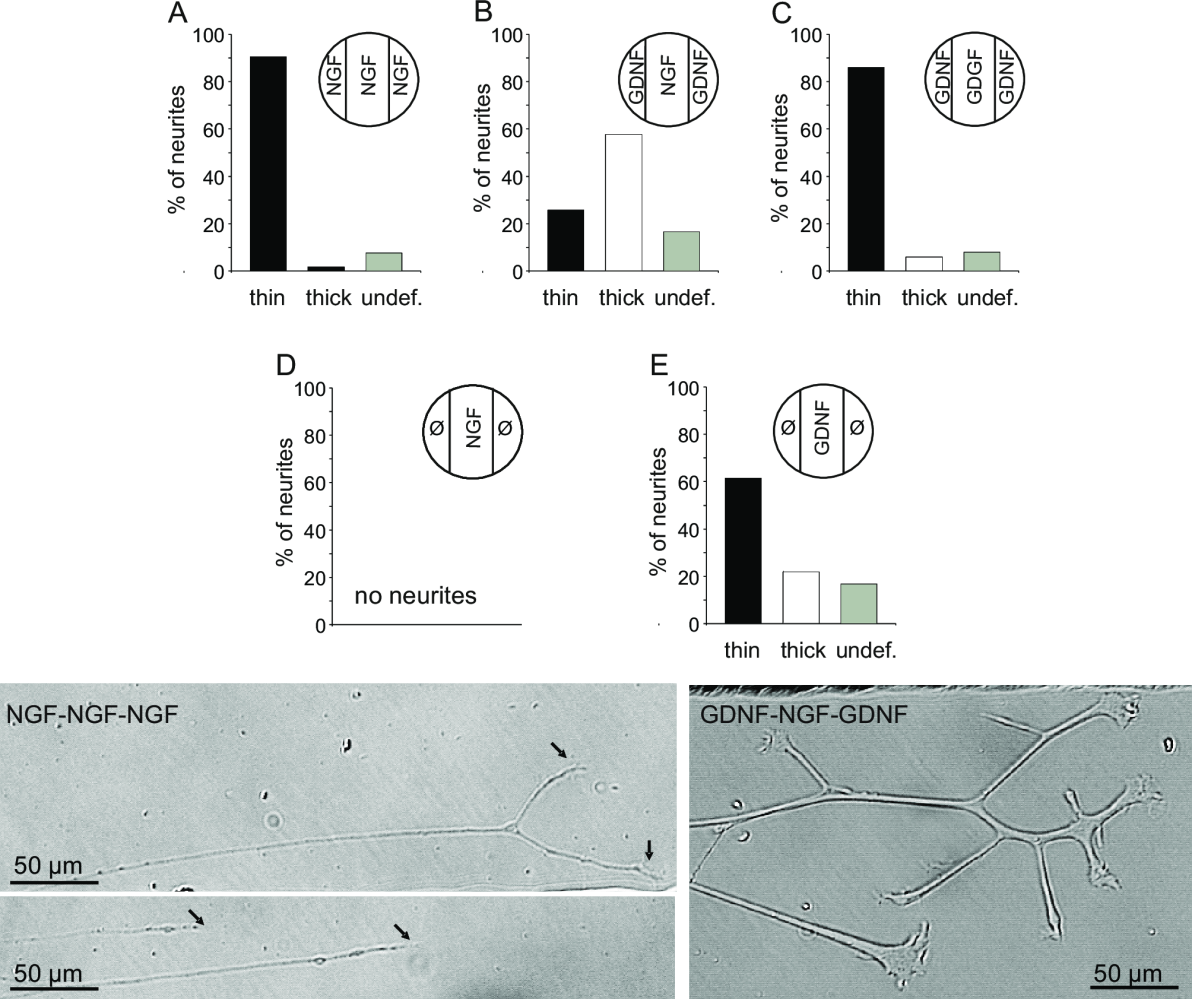
Morphology of neurite endings grown under different growth factor conditions. Top: Percentage of neurites with only thin endings (black bars), with one or more thick endings (grey bars), and undefined (white bars) under different culture conditions A—E. Insets show the allocation of growth factors in the compartments of the culture chamber. Qualitative visual determination was done from microphotographs taken after 4 to 5 days in culture. Abbreviations indicate the growth factors (GF) in the chambers, e.g., G-N-G denotes GDNF in the lateral compartments and NGF in the central, somata-containing compartment. Bottom: Examples for thin (left) and thick (right) neurite endings are shown in microphotographs. Media conditions in the compartments as indicated. Number of animals: 3 to 5 for A—E, respectively.

To evaluate neurite ending morphology, microphotographs from all neurites were taken after 4–5 days in culture. From these photographs, neurite endings were visually classified categorizing into those with thin growth cones, with pronouced growth cones (see Fig 2), or those that could not be clearly classified, showing only slightly thickened endings.

For quantification of CGRP release per length of neurite, microphotographs were taken after 7 days in culture directly before experiments. Total length of neurites was measured from all outgrown neurites using ImageJ [https://imagej.nih.gov/ij] by tracing with a pointing device.

For the neurite ending morphology investigation and the CGRP release experiments, different sets of cultures were used.

### CGRP release experiments

Neuropeptide release experiments were conducted at 37°C after 7 days in culture. After washing all three compartments twice with external solution containing (in mM) NaCl 145, KCl 3.5, MgCl_2_ 1, CaCl_2_ 2, d-glucose 10 and HEPES 10, pH 7.4, compartments were first incubated for 5 min in 150 μl external solution. Supernatant (100 μl) was taken from each compartment separately to determine basal CGRP release (see Table 2). This was followed by a 5 min stimulation period, applying 150 μl external solution containing either high potassium (60 mM, equimolar exchange with NaCl) or capsaicin (300 nM) either to the central or to both side compartments with the other compartment(s) receiving plain external solution. Incubation fluid was again sampled separately as above. Two final 5 min incubation periods in external solution were followed to assess reversal of stimulated CGRP release. The incubation fluid was stored at minus 25°C until the CGRP content of the incubation eluate was measured offline using a commercial EIA kit (Bertin Pharma, Montingy le Bretonneux, France) with a detection limit of 2 pg/ml. The antibodies used are directed against human α/β-CGRP. The EIA plates were determined photometrically using a microplate reader (Dynatech, Channel Islands, UK) [25] [34].

**Table 2:** Basal CGRP secretion; n: Number of compartments/animals; values represented as pg/ml (mean ± SEM).

	N-N-N condition	G-N-G condition	Ø-G-Ø condition
neurite compartments	7.1 ± 1.6, n = 20/5	17.2 ± 1.4, n = 29/7	11.6 ± 1.4, n = 29/5
somata compartments	84.12 ± 21.7, n = 8/5	146.7 ± 17.7, n = 12/4	115.9 ± 17, n = 13/5

Only chambers with ≥ 7 lanes with outgrown neurites in each side compartment were used for evaluation of CGRP release and are included in the data set. This selection criterion was chosen because 7 and 8 lanes of outgrown neurites per compartment were sufficient to detect a significant increase in CGRP content after stimulation with high potassium or capsaicin solution (individually tested for all chambers with 7 or 8 lanes, n = 9, p = 0.02 Wilcoxon test; 8 lanes were included because the number of 7 lanes was too low for statistical testing).

In our setup, basal CGRP release could possibly be influenced by mechanical stress induced by changing the solutions during the experiment. To reduce variability, the data were referred to the individual baseline value. This value was subtracted from all four data points of an experiment. Thus, only the absolute change in CGRP release (Δ pg/ml) is displayed in the figures showing the time course of stimulated CGRP release.

Table 2 depicts the values of basal CGRP secretion measured in the first eluation step before potassium stimulation. Values from neurite and somata compartments were pooled, separately, regardless of whether or not they were subsequently stimulated. The group Ø-N-Ø was not tested for basal CGRP release because there was no neurite outgrowth.

### Chemicals

All chemicals were obtained from commercial sources as given above (in brackets).

### Statistical analysis

The number of lanes occupied by outgrown neurites and total length of neurites in the different experimental groups were compared using a one-way analysis of variance (ANOVA) followed by Fisher’s least significant difference (LSD) test. For the CGRP release experiments, in all experimental groups data were analyzed for the effect of stimulation (high potassium or capsaicin) as compared to baseline values using the nonparametric Wilcoxon matched pairs signed–rank test to test whether increases in CGRP release were significant. The overall stimulated release was calculated as follows, the values of the stimulated and the two successive decay samples were added up, and the baseline values measured in the first eluation step were multiplied by three and subtracted to gain a quasi-area under the curve (AUC). Finally, to achieve group comparison, the AUC values were normalized to the total neurite length (in mm, measured in microphotographs taken from each chamber) in both neurite compartments (AUC/mm, given as mean ± SEM). Normalized data were entered into a one-way ANOVA followed by LSD test. To compare CGRP release by high potassium and capsaicin stimulation, we conducted a general linear model analysis (GLM) together with a post-hoc test (Fisher`s LSD). Only compartments with 7 or more lanes with outgrown neurites were analyzed. Data are displayed as mean ± SEM, statistics were calculated with Statistica 7 software (StatSoft, Tulsa, USA) and differences were considered significant at p <0.05 and are marked by asterisks.

## Results

### The morphology of outgrown neurite endings of cultured porcine DRG neurons is affected by growth factors

DRG somata cultured in the central compartment of a three-compartment Campenot chamber are spatially separated from their outgrown neurites in the adjacent lateral compartments. A fluid-tight separation of the different compartments is established by the silicon grease seal, allowing for exposure to different combinations of growth factors between compartments (Fig 1).

Here, we analysed the ending morphology of neurites grown in the lateral compartments with different growth factor combinations. Regularly, the first neurites reached the lateral compartments about 3 days after somata plating. Examination 4–5 days after plating showed differently shaped terminals. When nerve growth factor (NGF) was added to the lateral compartments (N-N-N condition, Fig 2 top A), out of 169 neurites, almost 90% showed only thin endings (example see Fig 2 bottom, left). Only 2% were classified as thick, having one or more of these growth cones (Fig 2 bottom, right); 8% could not be clearly allocated to either group. As previously shown, NGF cultured somata do not develop neurites growing into the lateral compartment without added growth factor there (Ø-N-Ø condition, Fig 2 middle D) [35]. However, when glial cell line-derived growth factor (GDNF) was present in the neurite compartment of NFG cultured somata (G-N-G condition, Fig 2 top B), surprisingly, we found that (i) there was a strong outgrowth of neurites and (ii) the proportions thick vs. thin changed considerably. Here, out of 260 neurites, 26% had only thin endings, but nearly 58% developed one or more thick growth cone(s). About 16% of the neurites showed a morphology which could not be classified into either ending type. To test whether GDNF presence at the neurite site per se induces growth of thick endings, we investigated also G-G-G condition (Fig 2 top C). Out of 217 neurites, 86% had only thin endings, 6% had thick endings, and 8% were undefined. In contrast to the Ø-N-Ø condition, we can show that GDNF cultured somata develop neurite outgrowth into the adjacent neurite compartment even when no growth factor was added there (Ø-G-Ø condition, Fig 2 middle E). Here, out of 160 neurites, 61% had only thin endings, but also a considerable number of neurites developed thick growth cones (22%) or could not be classified into either of the main groups (17%).

### The number and total length of cultured outgrown neurites is affected by growth factors

**Fig 3:**
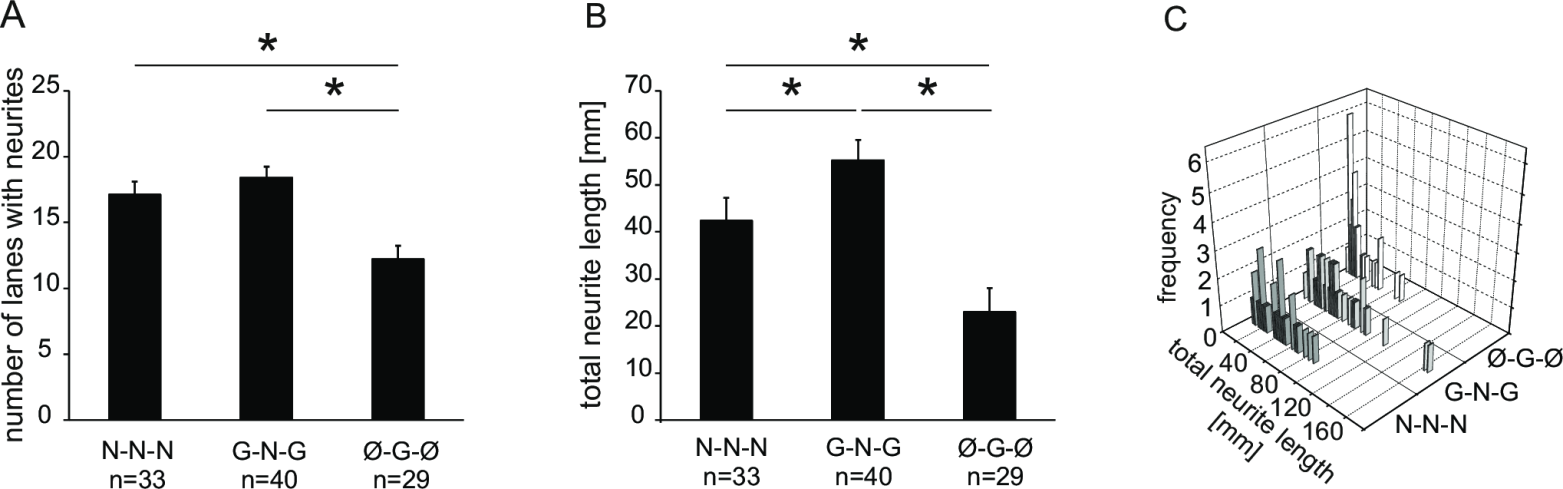
Number of lanes with outgrown neurites in the lateral compartments and total neurite length in the presence of different growth factors. (A) Number of lanes with outgrown neurites per lateral compartment. (B) Total length of the neurites per lateral compartment measured from microphotographs. (C) Frequency distribution of total neurite length for all single lateral compartments. Single data same as in (B). Error bars indicate mean ± SEM; asterisks indicate p<0.05. Number of animals: N-N-N: 7; G-N-G: 10; Ø-G-Ø: 5.

We next investigated whether the growth factors in the side and central compartments affect also the number of outgrown neurites. We took microphotographs of all outgrown neurites after 7 days in culture. The culture chambers used were the same as those used for the subsequent CGRP experiments on the same day (see below). For NGF cultured somata, NGF or GDNF in the side compartment did not make a significant difference regarding number of lanes with outgrown neurites (17.2 ± 0.9 lanes, n = 33 vs. 18.4 ± 0.8 lanes, n = 40; Fig 3A). For GDNF in comparison to NGF cultured somata and no growth factor in the side compartment, the number was significantly lower (12.2 ± 1 lanes, n = 29; one-way ANOVA F(2;99) = 11.758; p<0.001; p<0.001 Ø-G-Ø vs. N-N-N and G-N-G, respectively). Although the number of occupied lanes between conditions N-N-N and G-N-G was not significantly different, visual inspection suggested that branching of the neurites seemed to be higher in G-N-G condition (not shown). Therefore, we also measured total cumulative length of neurites (Fig 3B). It was 42.5 ± 5 mm per lateral compartment for N-N-N condition (n = 33). For G-N-G condition, the total length was significantly higher with 55.3 ± 4.3 mm (n = 40; one-way ANOVA F(2;99) = 11.663; p<0.001; p = 0.05 LSD post hoc test). For GDNF cultured somata with no growth factor in the neurite compartment, the mean total length was significantly lower with 22.9 ± 5 mm (n = 29; p<0.001 Ø-G-Ø vs. N-N-N and G-N-G, respectively; both LSD post hoc test). The distribution of neurite length for the individual compartments and for the different culture conditions shows comparably higher number of compartments with short total length in the experiments with Ø-G-Ø condition (Fig 3C).

### CGRP release from cultured neurites and somata under different growth factor conditions

Next, we investigated potassium-evoked CGRP release under three different culture conditions: N-N-N, G-N-G, and to cover GDNF responsive somata, we used the condition Ø-G-Ø. We know, that under Ø-N-Ø condition there is no neurite outgrow, therefore under Ø-G-Ø condition, we had the highest discriminatory power to have only neurites from GDNF dependent somata.

**Fig 4:**
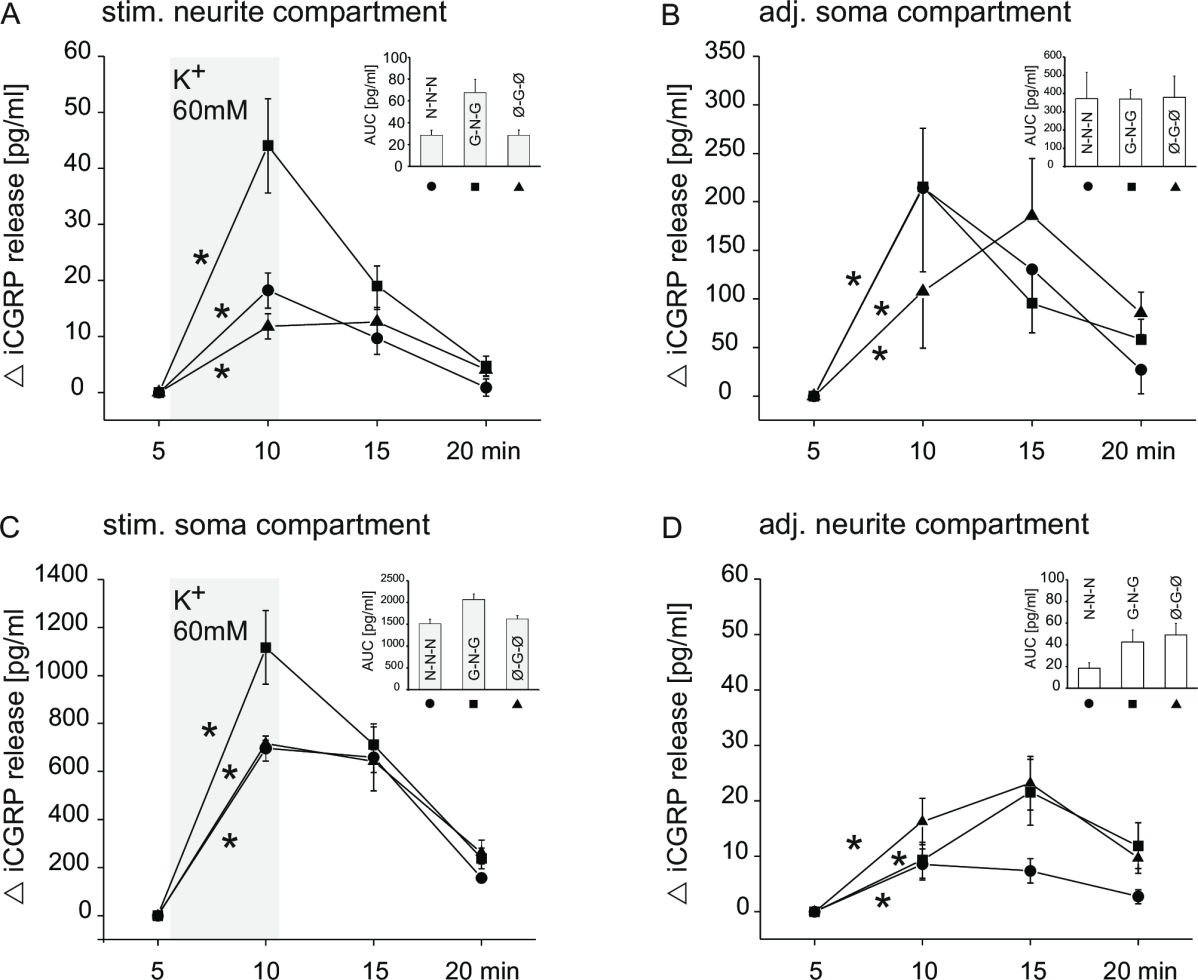
Time course of potassium-induced CGRP release in medium with different growth factors. CGRP release from potassium-stimulated neurites (A) or somata (C) and release in the adjacent soma (B) or neurite (D) compartment(s). (A) CGRP release in neurite compartment in response to potassium (60 mM) for 5 min (grey shading) and (B) concomitant CGRP release in the unstimulated adjacent soma compartment. (C) CGRP release in the soma compartment in response to potassium (60 mM) for 5 min (grey shading) and (D) concomitant CGRP release in the adjacent neurite compartment. Insets: Total CGRP release (as AUC (pg/ml)) determined from the respective time course experiments, grey columns indicate total CGRP release from the stimulated compartment(s), white columns from the adjacent one(s). Symbols and abbreviations: Circles denote NGF in the lateral and central compartments (N-N-N); squares denote GDNF in the lateral compartments and NGF in the central compartment (G-N-G); triangles denote no growth factor in the lateral compartments and GDNF in the central compartment (Ø-G-Ø). A: ● n = 11/3, ■ n = 21/4, ▲ n = 18/3, B: ● n = 4/3, ■ n = 8/4, ▲ n = 8/3, C: ● n = 4/2, ■ n = 4/3, ▲ n = 5/2, D: ● n = 9/2, ■ n = 8/3, ▲ n = 11/2,(n = number of compartments/animals used). Error bars indicate ± SEM, asterisks indicate p<0.05.

First, we had to verify that the quantity of CGRP released from the outgrown neurites in the side compartment was sufficient to be reliably detectable. We defined a cut-off criterion of at least 7 lanes with outgrown neurites per side compartment for inclusion into the data set (see Methods). To evoke CGRP release, high potassium solution (60 mM) was applied to the neurite compartment under all three culture conditions used. As shown in Fig 4A, potassium evoked a significant and reversible increase of CGRP into the eluate under all culture conditions (N-N-N: p < 0.001, n = 11 (number of compartments); G-N-G: p<0.001, n = 21; Ø-G-Ø: p<0.001, n = 18; all Wilcoxon test). Moreover, in the same experiments, we could detect a significant, reversible augmentation of CGRP content in the adjacent somata compartment that had not been chemically stimulated (N-N-N: p = 0.04, n = 4; G-N-G: p = 0.01, n = 8; Ø-G-Ø: p = 0.02, n = 8; all Wilcoxon test; Fig 4B). This indicates that a neuronal signal either by a wave of depolarization or by a train of evoked action potentials was transmitted retrogradely via the neurites, which provoked release of CGRP, most likely from the respective somata.

In a separate set of experiments the stimulation pattern was reversed. Now, the somata in the central compartment were stimulated with high potassium solution (60 mM). As expected, a significant and reversible release of CGRP into the eluate was observed (N-N-N: p = 0.02, n = 9; G-N-G: p = 0.017, n = 8; Ø-G-Ø: p < 0.01, n = 11; all Wilcoxon test; Fig 4C). Likewise, activation of the somata was followed by a significant increase of CGRP in the adjacent neurite compartments (N-N-N: p = 0.04, n = 4; G-N-G: p = 0.05, n = 4; Ø-G-Ø: p = 0.04, n = 5; all Wilcoxon test; Fig 4D). This indicates that again an electrical signal is propagated from the somata into the outgrown neurites in the lateral compartment.

Total amounts of CGRP release expressed as AUC values are shown in the inserts of Fig 4A–4D. With direct soma stimulation, the AUC values are considerably higher in comparison to those with indirect stimulation (Fig 4C and 4B). This is not surprising, as with indirect stimulation only those somata are activated which have outgrown neurites in the neurite compartment.

**Fig 5:**
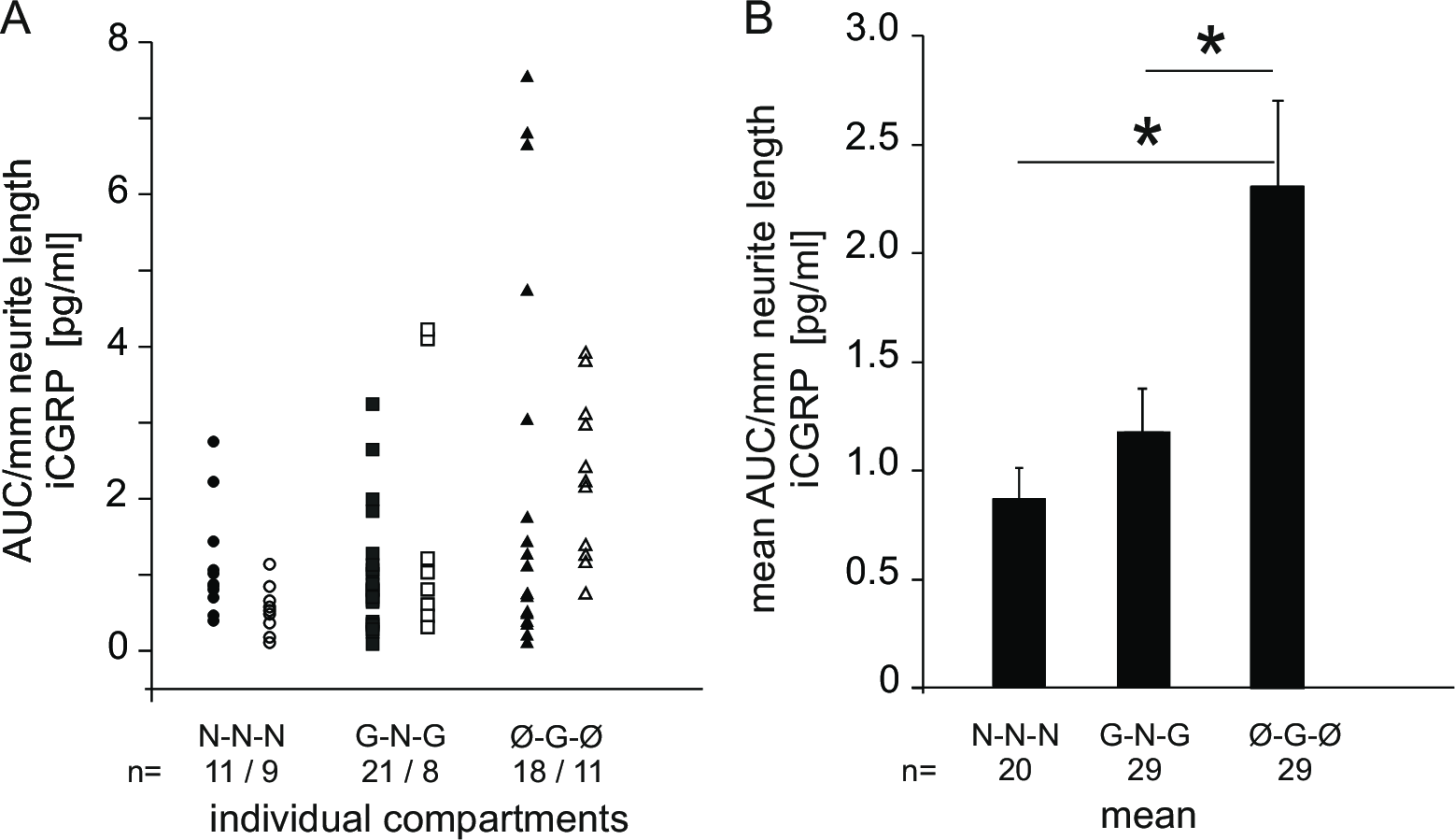
Total CGRP release (AUC) per mm neurite length. (A) Values for the individual compartments following neurite stimulation (filled symbols) and soma stimulation (open symbols). Symbols indicate the added growth factors in the chambers; N-N-N denotes NGF in the lateral and central compartments (circles); G-N-G denotes GDNF in the lateral compartments and NGF in the central compartment (squares); Ø-G-Ø denotes no growth factor in the lateral compartments and GDNF in the central compartment (triangles). Data derived from the experiments presented in Fig 4. (B) Mean values from data presented in A. Error bars indicate ± SEM; asterisks indicate p<0.05.

To account for the different total length of neurites under different culture conditions (see Fig 3B), the CGRP release was normalized. Fig 4 shows the time course of CGRP release expressed as pg/ml eluate regardless of the length of neurites involved; in the inserts, the AUC values are presented. To normalize the CGRP release for neurite length, we divided the AUC value per compartment by the respective overall length of the neurites. Fig 5A depicts the values for individual neurite compartments, either stimulated directly (filled symbols) or indirectly, when the somata were stimulated in the adjacent compartment (open symbols). Compartments are the same as presented in Fig 4A and 4C. In comparing the different growth factor conditions, we pooled the data from direct and indirect stimulation, as these were not significantly different. The averaged data are shown in Fig 5B. There were significant differences both between N-N-N condition and Ø-G-Ø and between G-N-G condition and Ø-G-Ø (one-way ANOVA F(2, 75) = 5.9, p<0.01; p<0.01 and p<0.001 for N-N-N and G-N-G vs. Ø-G-Ø, respectively). The normalization for neurite length reveals that the neurites from GDNF cultured somata without growth factor in the side compartment provide the highest relative CGRP release. Neurites from NGF cultured somata show no significant difference, regardless of the growth factor they are cultured in.

### GDNF and NGF cultured neurites express TRPV1, as indicated by CGRP release

**Fig 6:**
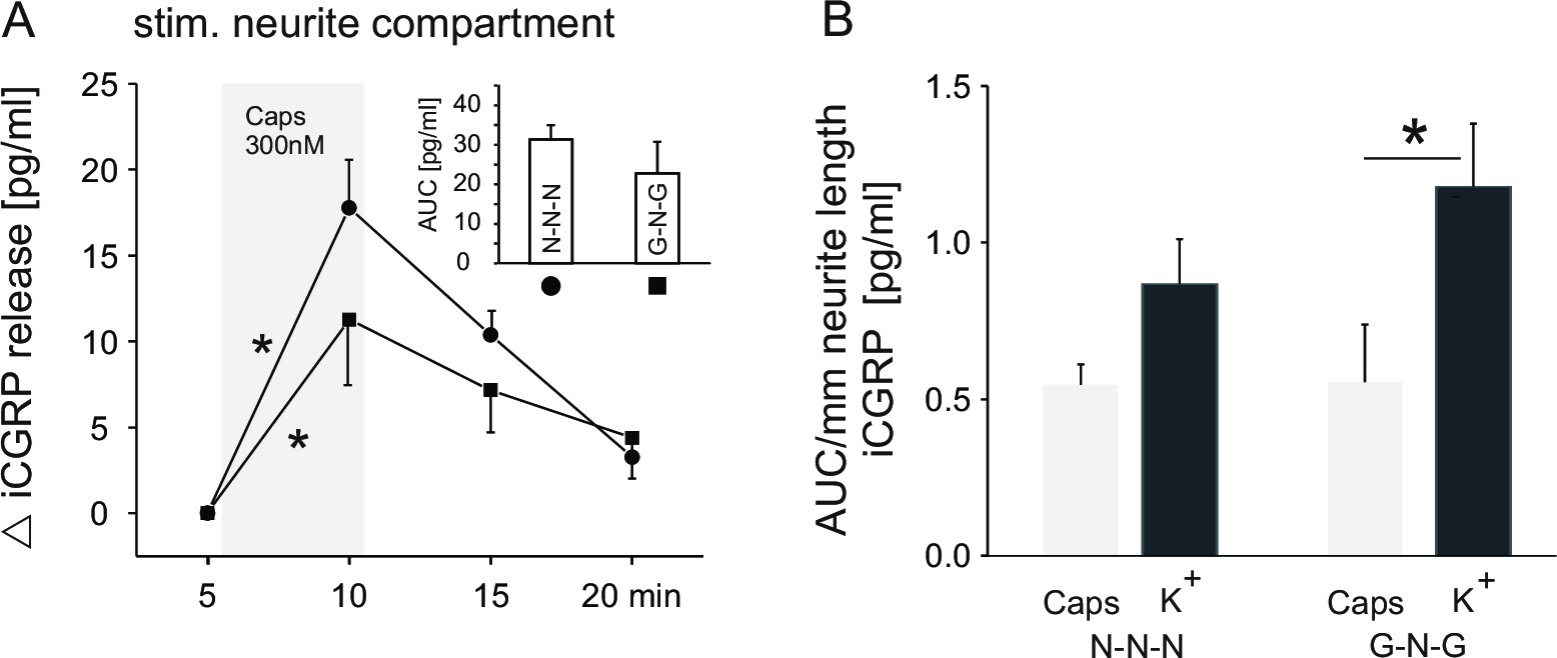
CGRP release from capsaicin stimulated neurites under different growth factor conditions. (A) Time course of CGRP release in response to capsaicin stimulation (300 nM) for 5 min (grey shading). Neurites were cultured with NGF (● n = 13/2 compartments/animals) or GDNF (■ n = 11/2). Somata were cultured with NGF in both cases. Inset: CGRP release as AUC (pg/ml) determined from the respective time course experiments. (B) Comparison between Capsaicin (300 nM, grey columns) and potassium (60mM, black columns) stimulated neurites, grown under NGF or GDNF, respectively. Somata were cultured with NGF in both cases. Data were taken from Figs 5B and 6A. Error bars indicate ± SEM. Asterisks indicate p<0.05.

It is well known that neurites from NGF-cultured somata are predominantly capsaicin sensitive nociceptors. We focused on this population of somata and questioned whether their neurites cultured in NGF or GDNF differ in capsaicin-evoked CGRP release. As reported above, when neurites from NGF cultured somata were grown in GDNF (G-N-G condition), we observed large growth cones and pronounced neurite branching, in contrast to NGF grown neurites. We therefore asked whether both groups of neurites respond to capsaicin (300 nM) as a specific stimulus for TRPV1, using CGRP release as readout. As expected, in NGF cultured neurites, capsaicin induced a significant and reversible increase of CGRP in the eluate (p<0.001, n = 13, Wilcoxon test, Fig 6A). Interestingly, also GDNF cultured neurites showed a significant and reversible increase of CGRP (p<0.001, n = 11, Wilcoxon test). When overall capsaicin-induced release (AUC, see insert Fig 6A) was normalized for total neurite length, no significant difference in release between neurite culture conditions was observed (Fig 6B, grey bars).

A group comparison between high potassium and capsaicin stimulation, assuming that both stimuli are maximal for NGF and GDNF cultured neurites (N-N-N vs. G-N-G), revealed that capsaicin evoked significantly less CGRP release than potassium in GDNF cultured neurites per mm total neurite length (GLM, LSD post-hoc test, p<0.03, Fig 6B).

## Discussion

### Growth factor-dependent neurite elongation and ending morphology

Using a three-compartment Campenot chamber, we investigated in a first step neurite outgrowth, elongation and ending morphology of NGF and GDNF cultured somata. We demonstrate that porcine NGF cultured DRG somata do not grow neurites into side compartments without added NGF (see Fig 2D, Ø-N-Ø condition). This has been shown before for newborn rat sympathetic neurons and adult rat DRG neurons [23]. In contrast, GDNF cultured somata send neurites into the side compartments even when no growth factor was added there (see Fig 2E, Ø-G-Ø condition). This suggests that activation of the GDNF receptor complex GFRα1/c-Ret in the somatic membrane is sufficient for both, neurite outgrowth from the somata and neurite elongation into the adjacent compartment; thus, GDNF in the lateral compartment is not mandatory for outgrowth. The compartmented culture chamber thus offers an *in vitro* method for functional sorting of subsets of neurites from NGF or GDNF cultured somata for further investigation. It could become an alternative method to sorting somata using magnetic beads [36] for consecutive cultivation and investigation of neurites.

*In vivo*, the composition of growth factors can change in the environment of neurite endings of sensory neurons after nerve injury or inflammation, resulting in hyperalgesia and pain [5,6,37,38]. With the focus on NGF cultured somata, presumably nociceptors, we used the compartmented culture chamber to model these changes. We cultured somata in NGF containing medium and added GDNF to the lateral compartments (G-N-G condition). Unexpectedly, there was a pronounced outgrowth into these compartments (see Fig 2B). Moreover, we observed a clearly more branched neurite network in the presence of GDNF compared to NGF (G-N-G vs. N-N-N). In the somata compartment, outgrowth and elongation of neurites is undoubtedly evoked by NGF/TrkA signaling. For the effect of GDNF in the side compartment we can only speculate: Neurite outgrowth into the side compartment may be stimulated via activation of GDNF binding sites at scattered Schwann cells associated with neurite endings. In Schwann cells, a GFRα1/c-Ret independent signaling receptor for GDNF is the neuronal cell adhesion molecule (NCAM) which can be co-expressed with GFRα receptors [39,40]. Binding to NCAM leads to activation of Fyn and FAK in the cytoplasma, a signaling pathway different from c-Ret [41]. Besides acting as a neurite guidance molecule, GDNF is able to stimulate Schwann cell migration via signaling through NCAM independent of c-Ret [40].

In comparing neurites under N-N-N and G-N-G culture condition, in addition to pronounced arborization in the latter, there was also a difference in growth cone morphology. Many neurites cultured with GDNF developed large growth cones compared to neurites cultured with NGF (see Fig 2, bottom). This suggests that cytoskeletal activity within the growth cone of neurites from NGF cultured somata, may diverge due to different intracellular signaling mechanisms induced by binding of either NGF or GDNF to their receptors [3,42–44]. Interestingly, under G-G-G condition, growth cone morphology closely resembled that of N-N-N condition (see. Fig 2) with only few thick endings growing out. Functionally, *in vivo*, changes in the composition of growth factors in the environment of a sensory neurite with subsequent morphological changes could support gain of function in sensory neurons. This is particularly true for skin innervation, as the epidermis has been shown to have higher levels of keratinocyte derived GDNF levels, whereas in the dermis fibroblast-derived NGF is dominating [45].

### Normalization of CGRP release from isolated neurites

In the present study, we were able to detect potassium (60 mM) -evoked CGRP release from only a few (≥7) spatially, but not functionally isolated neurites using the Campenot chamber system. The specificity of stimulus-evoked CGRP release was indicated by a significant and reversible increase of CGRP in the eluate (see Fig 4A). To quantify and compare CGRP release under different growth factor conditions, we normalized total CGRP release to overall neurite length (AUC/mm). This seems to be warranted, as experiments using isolated skin or sciatic nerve preparations [46–49] suggest that CGRP is not only released from terminals but also along the axons. We have shown previously that isolated sciatic or vagus nerves release CGRP upon stimulation in a calcium-, receptor- and concentration–dependent manner and also demonstated that a classical exocytosis of peptide-filled vesicles occurs along the axonal membrane [48,50,51]. Thus, we assume for the present preparation, that both terminals and neurites along their length contribute to CRGP release.

### In the pig, GDNF responsive neurons are peptidergic

Using the Campenot culture system, we show that high potassium application not only evokes CGRP release from somata and neurites of NGF cultured somata (N-N-N condition), but surprisingly also from GDNF cultured ones (Ø-G-Ø condition) (see Fig 4A and 4C). In neurites, the amount of CGRP released per mm is even significantly higher under Ø-G-Ø condition compared to N-N-N condition (see Fig 5B). Our finding that GDNF cultured porcine neurons are peptidergic is corroborated by immunoreactive (IR) staining experiments on porcine DRG somata. In lumbar DRG somata, 56% are reported to bind to the plant lectin B4 (IB4), which in mice is indicative for GDNF responsive neurons. Out of these, 72% are also CGRP-IR; vice versa, 65% of CGRP-IR lumbar DRG somata also express IB4, indicating that a high percentage of IB4 binding DRG somata in the pig is peptidergic [52]. These findings are in contrast to mice, where GDNF responsive neurons are mostly non-peptidergic [4,53,54]. Only about 4% of the IB4 binding neurons from adult mice are CGRP-IR and vice versa [55]. In rat, the co-localization of IB4 binding and CGRP-IR seems to be higher, about 30% of the IB4 binding somata are also CGRP-IR and, vice versa, 45% of the CGRP-IR somata are also IB4 binding [56].

Exocytosis of neuropeptide vesicles is dependent on increase in intracellular calcium concentration [57]. This increase can be initiated by activation of different voltage-gated sodium channels with subsequent activation of voltage-gated calcium channels. Contribution of N- and L-type calcium channels to neuropeptide release from sensory neurons has been affirmed using different tissue preparations [58–61]. The role of low threshold T-type channels is still a matter of discussion, maybe also due to lack of specificity of the classical T-type blocker Mibefradil that is mostly used in these studies [49,62,63]. The differences in CGRP release after potassium stimulation we found in neurites of NGF and GDNF cultured somata in the present study could possibly be due to the expression of different sodium channel isoforms in these two populations: Using the same culture chamber system, we found by calcium imaging experiments, under N-N-N and Ø-G-Ø culture conditions different proportions of neurites with TTX-resistant sodium channels [32]. In support of this, in mice it has recently been shown that different sodium channel isoforms contribute differentially to stimulated CGRP release from skin [64].

### CGRP increase in unstimulated compartments indicates an electrical signaling between neurites and respective somata

In our compartmentalized chamber system, application of high potassium solution in the neurite compartment not only released CGRP there, but also led to a significant and reversible release in the adjacent somata compartment (see Fig 4A and 4B). Reversing the stimulation pattern, signal propagation also takes place with potassium stimulation of the somata to the outgrown neurites in the adjacent compartments (see Fig 4C and 4D). This strongly indicates an electrical signal transmission via neurites to their somata and vice versa. According to the constant field equation [65], the application of high potassium solution induces a depolarizing shift of the membrane potential. In our experimental setup, an electrical signal must be transmitted either as depolarization wave or as trains of action potentials. Such signal transmission between neurite and soma is supported by our previous work with electrical stimulation of the soma compartment with subsequent calcium signals in neurites in the lateral compartment [32]. Moreover, we could show in co-culture of neurites and keratinocytes, that potassium stimulation of the somata induced, via release of a chemical mediator like CGRP, a calcium signal in the keratinocytes [31].

In the study presented, the amount of CGRP release from neurites is similar both for direct neurite stimulation and indirect stimulation in the soma compartment (see Fig 4A and 4D). In contrast, for somata, with neurite stimulation, presumably only their linked somata do release CGRP, whereas with direct stimulation all somata are stimulated. This difference in number of activated somata is reflected in the about five-fold difference in total CGRP release (see Fig 4B and 4C).

### Exposing neurites from NGF cultured somata to GDNF, the CGRP release is lower when stimulated with capsaicin, compared to potassium

We simulated environmental changes for neurites of NGF responsive somata by culturing them in GDNF (G-N-G condition), comparing to the N-N-N condition. When stimulated with high potassium, there was no significant difference in the normalized CGRP release per mm neurite length (see Fig 5B). Thus, extensive branching and thick growth cones under G-N-G condition do not seem to bear upon CGRP release. Potassium application leads to indiscriminative depolarization of all neurons. To target nociceptive neurons, we applied capsaicin (300 nM) to activate TRPV1 and induce CGRP release [66]. In isolated sciatic nerves, when stimulated both with low and high concentration of capsaicin under TTX, lidocaine, and Na^+^-free extracellular conditions to block action potential generation, no significant inhibition of CGRP release could be observed [49]. Therefore, although we did not block action potential generation, the high calcium conductance of the receptor is likely to be sufficient to induce CGRP release [67].

Similar to potassium stimulation, no difference in CGRP release was observed between N-N-N and G-N-G condition (see Fig 6B, grey bars). However, when neurites were cultured in GDNF, a group comparison between capsaicin and potassium stimulation showed a significantly lower CGRP release (AUC/mm) with capsaicin stimulation, which difference was less and not significant with NGF in the lateral compartments (see Fig 6B). For mouse DRG neurons it has been shown that both NGF and GDNF cause sensitization by increasing the capsaicin-evoked inward current, due to enhanced translocation of TRPV1 receptors into the neuronal plasma membrane [68–70]. An explanation for the difference in CGRP release between potassium and capsaicin stimulation could be that GDNF induces less expression of TRPV1 and translocation into the membrane than NGF.

## Conclusion

In summary, our data suggest that the compartmentalized Campenot chamber is a suitable system to investigate spatially but not functionally isolated neurite endings of neurotrophic- specific subgroups of DRG neurons. We show that local presence of NGF or GDNF in the environment of sensory nerve endings can contribute to structural and functional modification. Depending on type of growth factor we show for porcine DRG neurons population differences regarding neurite ending morphology. Moreover, we could detect stimulus-evoked neuropeptide release from only a few neurites of growth factor defined populations. We show that—in pig—not only NGF responsive neurons, but also GDNF responsive ones release CGRP. The system employed could be a model for studies addressing mechanisms associated with sensitization of functionally sorted nociceptive neurons.
